# The changing relation between alcohol and life expectancy in Russia in 1965–2017

**DOI:** 10.1111/dar.13034

**Published:** 2020-01-18

**Authors:** Inna Danilova, Vladimir M. Shkolnikov, Evgeny Andreev, David A. Leon

**Affiliations:** ^1^ Laboratory of Demographic Data Max Planck Institute for Demographic Research Rostock Germany; ^2^ International Laboratory for Population and Health National Research University Higher School of Economics Moscow Russia; ^3^ Department of Non‐communicable Disease Epidemiology London School of Hygiene and Tropical Medicine London UK; ^4^ Department of Community Medicine UiT Arctic University of Norway Tromsø Norway

**Keywords:** alcohol consumption, mortality, Russia

## Abstract

**Introduction and Aims:**

In the 1990s, a strong inverse relationship between life expectancy (LE) in Russia and mortality from alcohol poisoning was observed. This association is remarkable as this cause accounts for less than 2% of deaths each year. It can be explained by treating the alcohol poisoning mortality as the best available measure in Russia of the population prevalence of harmful drinking in any year which in turn associated with mortality from a wide range of causes. This study analyses the evolving relationship of LE with this mortality‐based measure of harmful drinking since 1965, and places it in a policy context.

**Design and Methods:**

We examine three periods: 1965–1984, a period of gradual LE decline; 1984–2003, a period of massive LE fluctuations; and 2003–2017, a period of LE improvement. Pearson's correlation coefficients and a linear relationship between annual changes in LE and alcohol poisoning were estimated for each period.

**Results:**

The strongest negative correlation between changes in LE and alcohol poisonings was found in 1984–2003. Over the period 2003–2017 a consistent positive LE trend emerged that was statistically independent of alcohol poisoning.

**Discussion and Conclusions:**

These results suggest that in the period from the mid‐2000s a growth of LE in Russia was to a large extent independent of changes in the population prevalence of harmful drinking. While there has been a reduction in mortality at ages 15–64, at older ages mortality reduction unrelated to alcohol has become an increasingly important driver of overall mortality improvements.

## Introduction

The problem of alcohol harm in Russia was recognised and discussed in the Soviet period [1, 2, 3]. Detailed quantitative evaluations were, however, not possible in this era as statistics on alcohol consumption and abuse were not publicly available from the early 1930s [4]. Moreover, data on mortality and causes of death were not published from the mid‐1970s [5]. This suppression of information meant that the full extent of the alcohol problem was not known [6]. It was only at the end of the 1980s that the necessary data became more readily available for research and analysis. Thus the scientific study of the problem of alcohol drinking in Russia was only able to develop from the mid‐1990s when the general health crisis became evident [7, 8, 9, 10, 11, 12].

The analysis of continuous mortality series including causes of death [5] showed that the low level of life expectancy (LE) in Russia and its further deterioration in the 1990s was caused mainly by excess mortality at working ages due to a range of causes especially those alcohol‐related and external causes [5, 8, 10, 13, 14, 15]. The same mortality components were seen to be important drivers of sharp LE fluctuations of the mid‐80s and the 1990s [10].

The first analysis of the remarkable correlation between LE and levels of alcohol consumption was published in July 1994 by Nemtsov and Shkolnikov in the Russian national daily newspaper *Izvestia*. Their article ‘Zhit “ili pit”?’ (‘To live or to drink?’) showed an almost perfect negative linear relationship between these two indicators [16]. Further studies confirmed a strong association between alcohol on one side and total mortality and LE on the other side [7, 11, 17, 18, 19].

Unfortunately, statistics on alcohol consumption in Russia are incomplete since they do not capture consumption of illegal and home‐brewed alcohol and non‐beverage alcohols such as medicinal tinctures. Nemtsov and other scholars have produced alternative estimates of the ethanol consumption in Russia [1, 4, 7, 11, 20, 21]. However, for our purposes these estimates are unsuitable as they involve an element of circularity being partly dependent on mortality from alcohol‐related causes.

Given the specific pattern of drinking in Russia (spread of binge drinking, the dominance of vodka and the use of non‐beverage alcohol [22]), the level of directly alcohol‐related mortality can be used as a reasonable proxy for the prevalence in the population of harmful drinking much of which will have been episodic in nature [22]. Of all alcohol‐attributable causes, acute alcohol poisoning is the one most obviously related to episodes of harmful drinking as by definition it is caused by fatally high blood alcohol concentrations.

In the present study, we re‐examine the link between mortality and alcohol in Russia from 1965 using the most recent available data including the period of sustained LE improvement from 2003 to 2017.

## Design and Methods

### Data

All‐cause mortality indicators and life tables for Russia for the period 1965–2017 were obtained from the Human Mortality Database [23]. Data for 2015–2017 were preliminary and obtained through personal communication with the Human Mortality Database team. Age‐specific mortality rates for alcohol‐related causes were obtained from the Russian statistical office. For the initial analysis, we used data on alcohol poisonings as our proxy measure for the prevalence of harmful drinking in the population in any 1 year. During the period 1965–2017 alcohol poisonings accounted for between 0.5% and 1.9% of total male aged standardised mortality rates and from 0.2% to 0.9% of the equivalent female mortality rates. In this context, alcohol poisoning also has the advantage as constituting only a small fraction of total mortality. Thus changes in mortality from alcohol poisonings cannot, for arithmetic reasons, alone produce any substantial effect on LE. On the other hand, it is still numerically large enough to not suffer from excessive random fluctuations.

For the sensitivity checks, we also used data on mortality from mental and behavioural disorders due to use of alcohol and alcoholic liver disease.

Detailed information on specific International Classification of Diseases codes used in our analysis and a commentary on the consistency of alcohol‐related causes in statistics is given in Appendix S1 (Supporting Information).

### Methods

We estimated the association between the time series of the life expectancy and series of the age‐standardised death rate (SDR) from acute alcohol poisoning in Russia using a ordinary least squares linear regression model of the first‐order differences. These differences are stationary both in males and females with the augmented Dickie‐Fuller test indicating the significance of *P* < 0.005.

The ordinary least squares regression model is:

(1) $$\Delta {\mathrm{LE}}_{t}=a+b\Delta {\text{AlcPois}}_{t}+\varepsilon_{t},$$

where *Δ*LE_*t*_ is the change in LE between years *t*−1 and *t* and *Δ*AlcPois_*t*_ is the corresponding change in SDR by alcohol poisonings per 100 000 person‐years. In addition to the regression, we estimate the Pearson's correlation coefficient (*r*) between annual changes in LE and changes in SDR by alcohol poisoning.

We estimate the parameters *a*, *b* and *r* for males and females and separately for the three time intervals reflecting different regimes of LE dynamics:

- 1965–1984—the period of gradual and continuous LE decrease.
- 1984–2003—the period of large‐magnitude fluctuations that began with the anti‐alcohol campaign of 1985.
- 2003–2017—the current period of continuous LE increase with an especially steep increase in 2005–2008.

We estimated the parameters of ordinary least squares‐regression model and *r* for the whole period 1965–2017.

The model (1) prediction of the annual change in LE is a sum of two components: the component depending on the change in SDR for alcohol poisoning ( *bΔ*AlcPois_*t*_ ) and component *a* expressing a constant annual change in LE that is not associated with the changes in mortality from alcohol poisoning.

In addition, we estimated how LE would evolve within each of the three time intervals if there was no independent (non‐alcohol) component and all changes in LE were predicted by changes in alcohol poisonings:

(2) $${\mathrm{LE}}_{t}={\mathrm{LE}}_{t_{0}}+b\left({\text{AlcPois}}_{t}-{\text{AlcPois}}_{t_{0}}\right),$$

where ${\mathrm{LE}}_{t_{0}}$ and ${\text{AlcPois}}_{t_{0}}$ are the values of LE and SDR from alcohol poisoning in the first year of the time interval (1965, 1984 or 2003), LE_*t*_ and AlcPois_*t*_ are the respective values in the current year *t*.

To see to what extent our results depend upon the choice of alcohol poisoning as a proxy for the prevalence of harmful drinking, we carried out a sensitivity analysis that included two additional models. In the first model, we replaced alcohol poisoning by the group of other causes directly linked to alcohol that includes ‘Mental and behavioural disorders due to use of alcohol’ and ‘Alcoholic liver disease’. In the second model, we combined alcohol poisoning with these two causes.

While earlier works [7, 18, 19] have suggested that changes in LE and changes in estimates of alcohol consumption are effectively instantaneous, we also estimated Pearson's correlation using lags of +1, +2 and +3 years.

## Results

**Figure 1:**
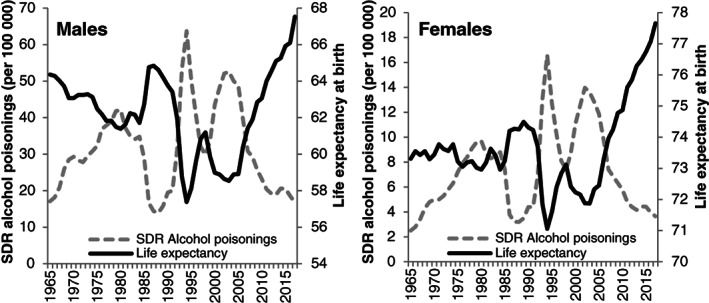
The ‘butterfly’ figure: Life expectancy and mortality from alcohol poisonings (standardised death rate [SDR] per 100 000) in Russia in 1965–2017.

Visual inspection of the association between LE and SDR for alcohol poisoning (Figure 1) shows a remarkable mirroring of the trends that can be described as having a ‘butterfly’ shape. However, since the late 2000s, the symmetry between the LE and the alcohol poisoning trends became less apparent. In particular, despite a slowdown in the decline in alcohol poisoning in 2010–2015, life expectancy continued to rise without interruption.

### Estimates of linear association

The relationships between changes in alcohol poisoning and in LE (Equation 1) are shown numerically in Table 1. The correlation is significant taken over the entire period under study (1965–2017). However, it was strongest in the period of large variation (1984–2003).

**Table 1:** Association of annual changes in the life expectancy at birth with age‐standardised death rates (per 100 000) from acute alcohol poisoning by sex and time period^a^

	1965–1984	1984–2003	2003–2017	1965–2017
*Males*				
Intercept	−0.058 (−0.184, 0.067)	−0.046 (−0.242, 0.150)	0.395 (0.099, 0.691)	0.060 (−0.044, 0.164)
Slope	−0.094 (−0.139, −0.035)	−0.129 (−0.152, −0.107)	−0.097 (−0.173, −0.020)	−0.132 (−0.150, −0.114)
Pearson's *r*	−0.65 (*P* = 0.003)	−0.95 (*P* < 0.001)	−0.62 (*P* = 0.02)	−0.90 (*P* < 0.001)
*Females*				
Intercept	0.077 (−0.066, 0.221)	−0.003 (−0.107, 0.113)	0.268 (0.112, 0.424)	0.089 (0.024, 0.154)
Slope	−0.289 (−0.551, 0.028)	−0.240 (−0.286, −0.194)	−0.205 (−0.362, −0.049)	−0.256 (−0.297, −0.215)
Pearson's *r*	−0.49 (*P* = 0.03)	−0.94 (*P* < 0.001)	−0.64 (*P* = 0.02)	−0.87 (*P* < 0.001)

a The 95% confidence limits of the intercept and the slope estimates and *P* values for the Pearson correlation coefficients are given in parentheses.

The regression slopes do not change substantially across the three periods with the confidence intervals (CI) overlapping each other. During the period 2003–2017, a decrease of one per 100 000 in the SDR for alcohol poisonings was associated with an increase of 0.1 years in LE amongst males and 0.2 years amongst females.

Unlike the slopes, the intercepts from the regression are not the same across the three periods (Table 1). In 1965–1984 and 1984–2003, they are small and do not differ significantly from zero. However, in 2003–2017, the LE increase in Russia is characterised by a positive and statistically significant (*P* < 0.05) intercept.

**Figure 2:**
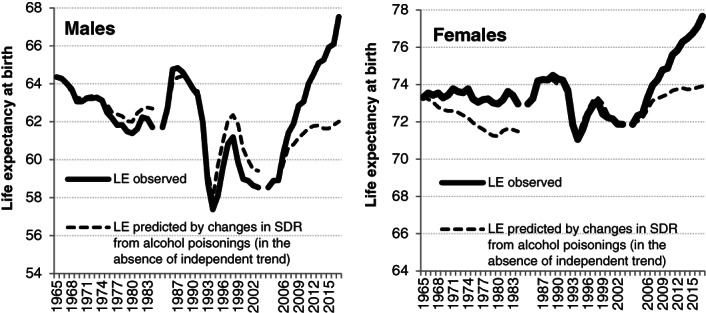
Observed changes in life expectancy (LE) as compared to changes predicted by mortality from alcohol poisonings for the periods 1965–1984, 1984–2003, 2003–2017. SDR, standardised death rate.

Figure 2 compares the observed trend in LE with that which would be observed if the only driver of LE change was alcohol as proxied by the SDR for alcohol poisoning (Equation 2). For males in 1965–1984 and 1984–2003, the accumulated gaps by the end of the period between the real change in LE and the change predicted by alcohol poisoning are quite small: 1.0 and 0.9 years for 1965–1984 and 1984–2003, respectively. But the same gap is much more substantial at the end of the 2003–2017 period. Indeed, the intercept of the linear model suggests a 5.4 year gain in the male LE by 2017. Another 3.5 years are associated with a reduction in mortality from alcohol poisonings.

For females, the linear regression model for the period 1965–1984 assumes that there was a small positive shift in LE that was independent of alcohol. While changes in alcohol poisonings predict 1.8 years loss in LE over that period, in reality it decreased by only 0.4 years. The estimates for the period 1984–2003 assume that loss in female LE in that period is almost entirely predicted by changes in mortality from alcohol poisonings. In the last period 2003–2017, similarly to males, the increase in female LE was substantially higher than the one predicted by changes in mortality from alcohol poisoning. Over the period 2003–2017, the annual alcohol‐independent increase (0.27 years) corresponds to 3.8 years of the female LE gain with the remaining 2.0 years attributed to alcohol.

Given the mortality levels from alcohol poisonings in the year 2017 and with the slope estimates for the period 2003–2017, we can estimate the lifetime that can be further gained if harmful alcohol consumption was eliminated: +1.6 years in males (95% CI 0.33, 2.9) and +0.7 years in females (95% CI 0.17, 1.3).

Results of our regression model suggest that in each of the periods a one‐unit change in mortality from alcohol poisoning is associated with a larger impact on LE amongst females compared to males. However, if instead of life expectancy we look at age‐specific death rates the greater female response disappears. For the period of the highest correlation, 1984‐2003, we estimated linear regression models predicting the changes in all‐cause mortality from the changes in mortality from alcohol poisoning by 5‐year age groups. In particular, we found that in all 5‐year age groups from 25–29 to 70–74 years, an increase of age‐specific death rates by alcohol poisoning by 1/100 000 predicted very similar male and female increases in the respective all‐cause death rates ranging from 8.5 (95% CI 6.3, 10.9) in males and 10.5 (95% CI 7.8, 13.2) in females at age 25–29 years to 74.8 (95% CI 47.9, 101.7) in males and 65.2 (95% CI 26.4, 104.0) in females at age 70–74 years.

Substantial difference between shapes of male and female survival curves (with higher values of the survival and the life expectancy functions in the female life table) constitutes the reason why the slopes do not differ significantly between sexes while calculated for mortality in age groups but are higher for females when it comes to LE.

It should nevertheless be noted that in reality male mortality from alcohol poisonings and changes in this mortality are much higher than those for females. In 1965–2017, the average annual change in SDR by alcohol poisoning was 3.93 male deaths per 100 000 compared with 1.05 female deaths per 100 000.

### Sensitivity analysis

The replacement of alcohol poisonings by a group of other alcohol‐related causes that includes mental and behavioural disorders due to use of alcohol as well as alcoholic liver disease reduced the predictive power of the model for the period 1984–2003 but increased it for the subsequent period of 2003–2017 (Appendix S2, Table S2a). The replacement by a larger group of alcohol‐related causes (alcohol poisonings, mental and behavioural disorders due to use of alcohol and alcoholic liver disease combined) results in slightly better predictive power of the linear models in the first and the third periods (Appendix S2, Table S2b). Nevertheless, our principal findings remain the same. Regardless of which group of alcohol‐related causes we use a significant constant term appears in the period 2003–2017, markedly distinguishing this period from the earlier periods of 1965–1984 and 1984–2003.

Testing the regression model with different time lags confirms that the association between changes in mortality from alcohol poisonings and LE is effectively instantaneous (Appendix S2).

## Discussion

In Russia, the strong correlation between annual changes in life expectancy and overall mortality on one side and annual changes in alcohol on the other side was first demonstrated by Nemtsov and Shkolnikov in the mid‐1990s. We have found that this relationship has changed over time. It was weaker before the beginning of the anti‐alcohol campaign of 1985, and it has also weakened in the last decade. In the recent period of health improvements, Russia experienced a substantial increase in LE that is not predicted by changes in the prevalence of harmful alcohol consumption as indexed by mortality from alcohol poisoning. The model for the period 2003–2017 suggests that even if mortality from alcohol poisoning stopped declining LE continues to increase. This implies that there are other factors not associated with changes in alcohol consumption that influence life expectancy in Russia. This has not been observed before. In the periods of 1965–1984 and 1984–2003 changes in mortality from alcohol poisoning predicted the overwhelming share of total changes in LE.

The appearance of the positive LE trend independent of alcohol poisoning may be the result of the recent decrease in mortality at old ages and its growing contribution to the changes in life expectancy at birth [24, 25, 26]. Since the late 2000s, the life expectancy changes in Russia are no longer driven only by mortality at young adult and midlife ages as it was in the 1970s to 1990s. The mortality decrease at old age is not a cohort but a period effect. In the mid‐2000s, mortality decline started simultaneously in all age groups. If it was a cohort effect, the mortality decline would occur progressively in successive age‐groups with time. Components and determinants of the Russian mortality reduction at old ages have been discussed elsewhere [24, 25]. In summary these include the modernisation of the health‐care system such as more widespread provision of acute cardiovascular services [27] and improvements in control of major risk factors including smoking and blood pressure. Despite the recent appearance of the positive alcohol‐independent trend in LE changes, the role of alcohol in Russian mortality over the past decade should not be underestimated. Our analysis showed that a large amount of the LE gained 2003–2017 was still associated with the decline in the prevalence of harmful drinking. A comparison with the other countries also shows that the role of alcohol in Russian mortality is still enormously high in the international context [28].

### Relationship to alcohol policy in Russia

A detailed analysis of the role of alcohol policy in driving the observed fluctuations in mortality from alcohol poisoning and other directly alcohol‐related deaths is beyond the scope of this paper. A considerable number of papers and books have been written about this issue [7, 21, 29, 30, 31, 32]. In summary, the downturn in alcohol‐related mortality in 1985 was the direct result of Gorbachev's anti‐alcohol campaign. Planning of anti‐alcohol measures started a number of years before Gorbachev came to power in 1985 [30] but the campaign was launched 2 months after he became the General Secretary. However, as the 1980s wore on, the attention of the Soviet state was drawn elsewhere, and this top‐down policy became increasingly ineffective and was effectively abandoned. This was followed by the collapse of the Soviet Union in December 1991 and a radical liberalisation of alcohol policies which led to cheap alcohol suddenly becoming widely available. The almost overnight abolition of state controls on the production and sale of alcohol, together with the emergence of countless small kiosks and other retail outlets in Russian cities selling cheap alcohol (some imported from Western Europe) coincided with the very fabric of Russian society falling apart. This included loss of employment, hyperinflation, wage arrears becoming common and the temporary collapse of law and order on the streets. The uncertainty and stress of these unanticipated and unwelcome changes as far as the bulk of the Russian population was concerned, led to a huge rise in the prevalence of harmful alcohol consumption. The subsequent emergence from the lowest point of life expectancy and maximum chaos in 1994 involved a degree of adaptation to the new post‐Soviet realities. However, there was a temporary setback in 1998, with the foreign currency crisis wiping out people's savings and associated security.

In terms of post‐Soviet alcohol policy, as early as May 2000 the Russian government set up a new regulatory agency, Rosspirtprom, which set about the transformation of the alcohol market through increased state control and the gradual elimination of small producers [18, 33]. In the mid‐2000s that these efforts become intensified when the Russian government realised that the scale of the continuing population losses at working age due to alcohol was becoming a barrier to economic progress. In 2005 a set of controls on alcohol production and sale was introduced [34] that had the important effect of dramatically accelerating the removal of smaller commercial alcohol producers from the market. Steps were also taken to ensure *denaturation* of various non‐beverage products containing ethanol such as hygiene substances, cleaning agents and eau de colognes [35]. The effectiveness of these policies is hard to evaluate due to absence of direct data connecting implementations of specific measures to mortality changes. However, it is notable that this does coincide with a sharp and persistent downturn in mortality from alcohol poisoning. More recent policy initiatives to increase the price of various beverages have been implemented, although in the most recent period some of these have been reversed presumably due to increasing economic difficulties in the country.

Changes in preferred beverage type are also important. Compared to the mid‐90s, the share of population consuming spirits has declined while the share of beer drinkers has increased [36, 37]. This happens partly because younger cohorts of Russians tend to drink less vodka and more beer compared to their older counterparts [37]. An analysis on married/cohabiting men aged 21–49 years, suggested a decline of average quantity of ethanol consumed on a typical day of consumption by about one‐third between 2002–2007 and 2008–2012 [38].

### Limitations

Real mortality from alcohol‐related causes can be under‐reported. Deaths from alcohol are often socially stigmatised [39, 40]. The relatives of the deceased may exert pressure on the doctors or forensic experts persuading them not to mention alcohol in the medical death certificate. It is possible that over time there have been changes in the willingness of experts to certify a cause as due to alcohol poisoning. The mortality trends in alcohol‐related causes can be also affected by changing approaches to selecting these causes as underlying. Some details on this issue are discussed in Appendix S1.

Finally, we would like to emphasise that our model is predictive but not causal. Though there are a number of micro‐level studies that confirm the causal relationship between harmful alcohol consumption and mortality in Russia, we undertook an analysis at the population level and did not control for any factors other than alcohol. Thus, we were unable to adjust for potential confounders. As commonly assumed, the increasing levels of alcohol consumption in Russia in the 1990s had been accelerated by social stress and economic insecurity after the collapse of the Soviet Union [41, 42]. Those factors and changes in them could influence mortality levels through other pathways.

## Conclusion

In this paper we have investigated the relationship between the trends of life expectancy and the mortality from acute alcohol poisonings as a proxy for the population prevalence of harmful drinking. We have shown that fluctuations in LE are strongly related to fluctuations in mortality from alcohol poisoning. However, the phenomenon of the overall low LE in Russia, versus its fluctuations, is complex and cannot be explained on the basis of alcohol alone. However, what we can say with some certainty looking at results of our models is that in the last decades of the 20th century the pattern of mortality change was established in Russia, with changes in the levels of harmful alcohol consumption being crucial in shaping the LE trend. Our analyses have shown that this pattern has recently changed. Factors not associated with the prevalence of harmful drinking have become more important for LE improvement. While alcohol still remains a major public health problem in Russia, other positive developments substantially contribute to the life expectancy rise.

## Supporting information


**Appendix S1**. Supporting information

**Appendix S2**. Supporting information
