# Supplementary material for: The changing relation between alcohol and life expectancy in Russia in 1965–2017
Source: Drug Alcohol Rev. 2020 Jan 18;39(7):790–6. doi: 10.1111/dar.13034 (PMC8607467; doi:10.1111/dar.13034)
Supplement: Supplementary file 1 — Appendix S1. Supporting information [file DAR-39-790-s001.docx]

**APPENDIX S1**

In the Soviet period, classifications of causes of death included “accidental poisoning by alcohol” as a separate category. It corresponded to the code E880 in the International Classification of Diseases (ICD)-7 and the code E860 in ICD-8 and ICD-9. ICD-10 as well as the (based on it) Russian Abridged Classification contains, besides the category “Accidental poisoning by and exposure to alcohol” (the code X45), two more items for alcohol poisonings: “Suicide by and exposure to alcohol” (X65) and “Poisoning by and exposure to alcohol, undetermined intent” (Y15). While the first added category is almost never used in Russia to code the underlying cause of death (only four deaths in 2017), selecting Y15 as an underlying cause has become more common in recent years. In 2000, alcohol poisonings with undetermined intent accounted for 4% among all alcohol poisonings in Russia. By 2017, this share increased up to 21%. Deaths from alcohol poisonings with undetermined intent are probably being used to mask mortality caused by alcohol since before 2015 the code “Y15” was not included into the group of alcohol-attributable causes in Rosstat reports [[43](#_ENREF_43)]. Thus, it is very likely that these deaths would otherwise be coded as accidental poisonings by alcohol. To keep the consistency in reporting of alcohol poisonings over time we aggregated ICD-10 codes “Y15”, “X65”, and “X45” into a single category “alcohol poisonings”.

Though there can be some discrepancies in how the intent of alcohol poisoning is determined, the fact of the poisoning per se is established based on the laboratory tests of blood alcohol concentration. Thus, we may hope that medical practitioners do not experience many difficulties while deciding whether alcohol poisoning should be certified as a cause of death, and the certification and coding approaches are stable in time. Still, some changes in the approaches and methods for the cause of death investigation could potentially affect the trend of mortality from alcohol poisoning. First, the methods for determining blood alcohol concentration have been developed since 1965. Next, the autopsy rate has been changing in Russia. Since the beginning of the 21st century, it has increased in Russia almost two-fold: from 33.1% in 2000 to 60.6% in 2017. Besides, in 2010 a new procedure of forensic medical examinations was introduced by the Ministry of Health and Social Development of the Russian Federation. In particular, it prescribes that blood and urine should always be checked for ethanol concentration in forensic examination except in cases of death of adults who have been in the hospital for a long time (more than 36 hours). The introduction of the new rules resulted in a sharp increase in the share of deaths with measurable levels of alcohol in blood between 2010 and 2011 – from 2.0% to 4.3%. We assume that it could not affect the trend of mortality from alcohol poisonings any significantly since it is unlikely that deaths with heavy alcohol intoxication could be overlooked by the experts and remain untested even before the new procedure was introduced. Also, no break in the series can be seen between 2010 and 2011.

It should be also noted that the structure of alcohol poisonings by the type of toxic substances has changed. In 2000, 80% of alcohol poisonings were specified as caused by the toxic effect of ethanol while the rest (20%) as of other and unspecified alcohols. By 2012, the share of ethanol poisonings gradually increased up to 90%. After that, it started to decline again and amounted to 84% in 2017.

The enlarged group of alcohol-related causes of death includes besides alcohol poisonings “Mental and behavioural disorders due to use of alcohol” (F10 in ICD-10) and “Alcoholic liver disease” (K70). The selecting of these causes of death as underlying probably more often depends on subjective views of a certifying doctor or a coder compared to alcohol poisoning. Thus, the mortality trends from these causes can be more severely affected by changing approaches to certifying and coding over time.
