# Supplementary material for: The changing relation between alcohol and life expectancy in Russia in 1965–2017
Source: Drug Alcohol Rev. 2020 Jan 18;39(7):790–6. doi: 10.1111/dar.13034 (PMC8607467; doi:10.1111/dar.13034)
Supplement: Supplementary file 2 — Appendix S2. Supporting information [file DAR-39-790-s002.docx]

**APPENDIX S2**

*Replacing alcohol poisonings with other alcohol-related* *causes*

Table S1a reports result from the OLS regressions and Pearson’s correlation coefficients between the changes in life expectancy and mortality from the group of alcohol-related causes that includes “Mental and behavioural disorders due to use of alcohol” and “Alcoholic liver disease”. The 95% confidence limits of the regression coefficients and *P*-values are given in parentheses.

**Table S2a. Associations between changes in the age-standardised death rates by the alcohol-related causes (other than alcohol poisonings) and life expectancy at birth by sex and time period**

|  | **1965-1984** | **1984-2003** | **2003-2017** | **1965-2017** |
| --- | --- | --- | --- | --- |
| ***Males*** | | | | |
| **Intercept** | -0.065 (-0.202, 0.071) | -0.016 (-0.363, 0.395) | 0.531 (0.359, 0.703) | 0.123 (-0.031, 0.278) |
| **Slope** | -0.344 (-0.597, -0.091) | -0.305 (-0.428, -0.183) | -0.283 (-0.410, -0.156) | -0.323 (-0.400, -0.247) |
| **Pearson’s r** | -0.57 (*P*=0.011) | -0.79 (*P*<0.001) | -0.81 (*P*<0.001) | -0.77 (*P*<0.001) |
| ***Females*** | | | | |
| **Intercept** | 0.018 (-0.097; 0.134) | 0.088 (-0.129; 0.305) | 0.378 (0.288; 0.468) | 0.122 (0.027; 0.217) |
| **Slope** | -0.871 (-1.58; -0.159) | -0.519 (-0. 75; -0.287) | -0.254 (-0.382; -0.127) | -0.481 (-0.621; -0.342) |
| **Pearson’s r** | -0.53 (*P*=0.019) | -0.75 (*P*<0.001) | -0.78 (*P*<0.001) | -0.70 (*P*<0.001) |

*Replacing alcohol poisonings with a larger group of alcohol-related* *causes*

Table S1b reports result from the OLS regressions and Pearson’s correlation coefficients between the changes in life expectancy and mortality from the enlarged group of alcohol-related causes. The 95% confidence limits of the regression coefficients and *P*-values are given in parentheses. The regression coefficients in Table S1a are consistent with those in Table 1.

**Table S2b. Associations between changes in the age-standardised death rates by the alcohol-related causes and life expectancy at birth by sex and time period**

|  | **1965-1984** | **1984-2003** | **2003-2017** | **1965-2017** |
| --- | --- | --- | --- | --- |
| ***Males*** | | | | |
| **Intercept** | -0.040 (-0.158, 0.079) | -0.013 (-0.209, 0.183) | 0.371 (0.138, 0.604) | 0.080 (-0.017, 0.178) |
| **Slope** | -0.087 (-0.131, -0.043) | -0.100 (-0.117, -0.083) | -0.092 (-0.141, -0.043) | -0.105 (-0.118, -0.091) |
| **Pearson’s r** | -0.71 (*P*<0.001) | -0.95 (*P*<0.001) | -0.76 (*P*=0.002) | -0.91 (*P*<0.001) |
| ***Females*** | | | | |
| **Intercept** | 0.102 (-0.029; 0.233) | 0.041 (-0.073; 0.154) | 0.266 (0.179; 0.353) | 0.104 (0.043; 0.165) |
| **Slope** | -0.323 (-0.536; -0.110) | -0.184 (-0.219; -0.148) | -0.173 (-0.235; -0.112) | -0.198 (-0.227; -0.169) |
| **Pearson’s r** | -0.61 (*P*=0.01) | -0.93 (*P*<0.001) | -0.87 (*P*<0.001) | -0.89 (*P*<0.001) |

*Introducing lags*

For the whole period 1965-2017, the Pearson’s correlation coefficients between the series taken with zero lags amounted to -0.90 in males and -0.87 in females. With the lag equal to one year, the *r* values diminish to -0.57 and -0.52, respectively. The lags of two and three years result in very low and statistically insignificant coefficients.
